# Nebulized colistin for treatment of ventilator-associated pneumonia caused by multidrug-resistant Gram-negative bacteria: we still need to straighten out the dose!

**DOI:** 10.1186/s13054-015-0966-x

**Published:** 2015-06-26

**Authors:** Patrick M. Honore, Rita Jacobs, Inne Hendrickx, Elisabeth De Waele, Jouke De Regt, Herbert D. Spapen

**Affiliations:** ICU Department, Universitair Ziekenhuis Brussel, Vrije Universiteit Brussel, 101 Laarbeeklaan, 1090 Jette, Brussels, Belgium

We applaud the extensive systematic review and meta-analysis by Zampieri and colleagues on inhaled antibiotics for ventilator-associated pneumonia (VAP) [1] but would like to add some comments regarding inhaled colistin for treatment of VAP caused by multidrug-resistant Gram-negative bacteria (MDR-GNB).

In patients with cystic fibrosis, the equivalent of 160 mg colistimethate sodium (CMS) produced high and therapeutic endobronchial concentrations [2]. However, to be effective in VAP caused by MDR-GNB, colistin must attain pulmonary concentrations that substantially exceed minimal plasma inhibitory levels for these microorganisms. Colistin aerosols used in the predominantly retrospective and observational studies summarized by Zampieri and colleagues contained CMS doses ranging from 80 to 300 mg [1]. These doses may be insufficient to grant any significant or additional therapeutic value to inhaled colistin, especially when used together with intravenous antibiotics. Indeed, a total daily inhaled dose of 240 mg CMS was found to be inadequate to treat lung infection caused by MDR-GNB [3]. A recent review recommended inhalation of 160 mg CMS every 8 h for severe pulmonary infections [4]. Finally, a daily dose of 1,200 mg inhaled CMS used in monotherapy or associated with a 3-day course of intravenous aminoglycosides cured VAP caused by MDR-GNB as effectively as VAP caused by β-lactam susceptible GNB [5]. In contrast with intravenous administration, high doses of nebulized colistin are not associated with an increased risk of kidney injury, even when given for a prolonged period of time [5].

We strongly salute aerosolized colistin as an important alternative or adjunct therapy to treat VAP caused by MDR-GNB, provided that the daily inhaled dose is sufficiently high.

## Abbreviations

CMS: Colistimethate sodium
MDR-GNB: Multidrug-resistant Gram-negative bacteria
VAP: Ventilator-associated pneumonia
